# Evolving treatment strategies in meningioma: from traditional approaches to emerging therapies

**DOI:** 10.1097/MS9.0000000000003556

**Published:** 2025-07-16

**Authors:** Tirath Patel, Fathimathul Henna, Hamza Yousuf Ibrahim, Arun Kumar Maloth, Aziz Ur Rehman, Syeda Ramish Zehra Kazmi, Abbas Hussain, Christopher Hanani

**Affiliations:** a Trinity Medical Sciences University School of Medicine, Saint Vincent and Grenadines; b Dubai Medical College for Girls, Dubai, UAE; c Jinnah Medical and Dental College, Karachi, Pakistan; d Kakatiya Medical College, Warangal, Telangana, India; e Liaquat National Hospital and Medical College, Karachi, Sindh, Pakistan; f Department of Neurology, Henry Ford Health, USA

**Keywords:** immunotherapy, meningioma, molecular therapy, surgical resection

## Abstract

**Background:**

Meningiomas are the second most prevalent adult central nervous system neoplasm, developing from arachnoid cap cells. While most are benign (WHO Grade I), atypical (Grade II), and anaplastic (Grade III), meningiomas portray aggressive behavior, higher relapse rates, and resistance to standard treatments.

**Objective:**

To study the progression of treatment strategies for meningiomas, underscoring emerging treatments and challenges, especially in recurrent and high-grade subtypes.

**Methods:**

A narrative review was conducted, identifying studies from the PubMed, Scopus, and Google Scholar databases (2000–2025). Keywords included “meningioma,” “surgery,” “radiotherapy,” “targeted therapy,” “immunotherapy,” and “PRRT.” Included studies addressed conventional or novel treatments for meningiomas.

**Results:**

Surgical resection continues as the gold standard, with the extent of removal impacting recurrence. Radiotherapy, comprising fractionated and stereotactic techniques, plays a vital role when surgery is incomplete or not possible. Chemotherapy demonstrates limited benefit. Advances in molecular profiling have shown genetic drivers (e.g., *NF2, TRAF7, AKT1*) and hormonal receptors as clinical targets. Emerging options include mTOR inhibitors (everolimus), anti-angiogenic agents (bevacizumab), PD-1/PD-L1 inhibitors (pembrolizumab), and peptide receptor radionuclide therapy (PRRT). Drug delivery treatments like convection-improved delivery show promise in improving CNS penetration. Nonetheless, resistance mechanisms, the absence of validated biomarkers, and insufficient trial data pose significant challenges.

**Conclusion:**

Management of severe aggressive meningiomas is evolving with novel molecular and immunotherapeutic strategies. A multidisciplinary treatment strategy combining surgery, radiation, and targeted therapies, facilitated by further clinical trials and biomarker research, is necessary to enhance patient outcomes.

**HIGHLIGHTS**

- Surgery and radiation still central: Surgical resection continues to be the gold standard for meningioma treatment, mainly for resectable tumors, while radiation therapy (FRT and SRS) is vital for inoperable or relapsing cases, although both have limitations in high-grade tumors.
- Emerging therapies provide promise: Targeted therapies (e.g., mTOR inhibitors, anti-angiogenics like bevacizumab) and immunotherapies (e.g., pembrolizumab) demonstrate early promise, especially for recurrent or dangerous meningiomas, but do not have long-term efficacy data and RCTs.
- Drug delivery innovation: Techniques like convection-enhanced delivery (CED) and intrathecal therapy target to surmount the blood-brain barrier, improving local drug concentration while decreasing systemic toxicity.
- Challenges with resistance and biomarkers: High relapsing rates, lack of understanding of resistance mechanisms, and poor validated biomarkers for treatment response continue to affect effective, individualized management.
- Research and trials needed: Large-scale, randomized, biomarker-driven clinical trials are necessary to confirm emerging therapies, refine combination regimens, and incorporate personalized medicine into standard care for aggressive and recurrent meningiomas.

## Introduction

Meningiomas are the second most common adult neoplasm of the CNS^[1]^. The arachnoid cells of the meninges, the brain’s outermost layer, are the source of these tumors. Meningiomas are often benign, non-invasive, and confined, but other meningiomas are more aggressive, with a high chance of recurrence, a tendency to invade the surrounding brain, and, in rare instances, extracranial metastases^[2]^. According to the WHO tumor classification system, meningiomas are divided into three grades: benign (Grade I), atypical (Grade II), and anaplastic (Grade III)^[1]^. Meningiomas that fall into the Grade I group are the most common (78–81%), followed by the atypical kind (15–20%), while just 1–4% are malignant or anaplastic^[2]^. Meningiomas have a yearly incidence of 2.3 per 100 000, rise with age, and peak in the seventh decade of life^[1]^. Meningioma is more common in women, most likely as a result of hormonal factors. Adults over 60 years of age had a greater prevalence rate^[3,4]^. Meningiomas, originating from the intracranial or spinal dura, have symptoms that depend on location; common signs include headaches, focal neurological deficits, and seizures. These tumors, often discovered incidentally, should be distinguished from gliomas, metastatic tumors, and other intracranial lesions^[5]^.

Radiation therapy is the first-line treatment for meningiomas that cannot be removed, while surgical excision is the main treatment for large, symptomatic, or observation failure tumors. Systemic salvage therapy is necessary for meningiomas that are progressive or recurrent^[6]^. Although the number of patients with recurring, progressive, and symptomatic meningiomas following repeated surgery and radiation therapy is modest, these patients pose a significant therapeutic challenge^[7]^. Potential therapies for recurrent meningiomas are being investigated through ongoing trials on VEGF-targeted therapies, and traditional cytotoxic agents have not proven effective. In Phase 2 trials, antiangiogenic therapies like antibodies and multi-tyrosine kinase inhibitors have demonstrated encouraging results. However, to validate these findings, prospective controlled trials are required. Immunotherapeutic approaches are being evaluated in ongoing national trials, and genetic studies have discovered novel targets^[8]^. So, emerging therapies require further research to establish their efficacy and safety in routine clinical practice.

This narrative review evaluates conventional and emerging treatments for meningiomas, identifying gaps in management, particularly in recurrent cases. It addresses key controversies, including the integration of novel targeted therapies, immunotherapy, and the personalized medicine landscape, to guide future research and clinical decision-making.

## Methods

This narrative review was conducted to identify the evolving treatment options for meningiomas, with a target on both traditional and emerging therapeutic approaches. The review underscored current clinical practices, ongoing research, and future directions in treating meningiomas, especially recurrent and high-grade subtypes.

### Literature search strategy

A comprehensive literature search was done using PubMed, Scopus, and Google Scholar databases, identifying studies published from January 2000 to March 2025. Keywords used in the search were “meningioma,” “intracranial tumor,” “meningioma surgery,” “surgical resection,” “radiotherapy,” “radiation therapy,” “antineoplastic agents,” “molecular targeted therapy,” “immunotherapy,” “mTOR inhibitors,” “anti-angiogenic therapy,” and “novel treatments.”

### Inclusion and exclusion criteria

Peer-reviewed studies, clinical trials, meta-analyses, and potential review articles written in English were included. Studies emphasizing the pathophysiology, genetic triggers, clinical presentations, and treatment results of meningiomas were included. Case reports, conference abstracts, non-English studies, and studies irrelevant to treatment strategies were excluded.

### Data extraction and synthesis

Two reviewers extracted the data, which included study design, sample size, intervention type, treatment outcomes, and safety profiles. Conflicts were solved through consultation with a third reviewer. The data were thematically analyzed to compare traditional treatments (surgery, radiotherapy, and chemotherapy) with novel treatments (targeted agents, immunotherapy, PRRT, and advanced drug delivery methods). Particular focus was ongoing clinical trials and studies involving high-grade or recurrent meningiomas.

## Background

### Pathobiology and genetic drivers

The development of meningiomas is through multiple genetic pathways. The most common genetic syndrome causing meningioma is Neurofibromatosis type 2 (NF2)-related Schwannomatosis^[9-11]^. It is a dominantly inherited disorder, mostly characterized by the development of schwannomas and meningiomas. Although NF2 is the most common mutation, there are related mutations, which are TRAF7, KLF4, AKT1, SMO, POLR2A, and PIK3CA, which are mostly presented as Grade I^[12]^.

Hormonal influences: Progesterone receptors (PR) exist in more than 60% of meningiomas^[13]^. There was a higher probability that these tumors were observed to be increased during pregnancy and hormone replacement therapy^[14,15]^. The expression of estrogen receptors (ER) and androgen receptors (AR) is very low. The expression of ER is very low^[16]^.

### Clinical challenges

Recurrence rates: Although meningiomas are considered benign tumors, the recurrence rate is higher^[17]^. In Grade I, there is a recurrence rate of around 10% after complete resection of the tumor; the most common site of the recurrence is the base of the skull due to residual tumor cells. In Grade II, there is a recurrence rate of 30–50% within 5 years; it has more aggressive tumor biology and excessive tumor invasiveness. In Grade III, there is over 80% recurrence due to the rapid progression of the tumor^[17]^. In conclusion, a higher recurrence rate remains a major clinical challenge, particularly in WHO Grade II and III meningiomas, where the current treatment options provide only temporary control. Future research must focus on biomarker-driven therapies and combination treatment strategies to improve long-term outcomes^[18,19]^.

Neurological symptoms: Meningiomas can lead to various neurological symptoms based on their size, location, and the extent of brain or nerve compression. These symptoms typically develop slowly over time, making early detection difficult. In patients with meningiomas, seizures often represent a significant clinical manifestation, highlighting the need for a thorough evaluation of underlying neurological, metabolic, and systemic contributors. Some of the common symptoms associated with meningiomas include dizziness, ataxia, mood disturbances, memory loss, seizures, headache, nausea, and vomiting. Neurological symptoms in meningiomas vary significantly based on tumor location, size, and growth rate. While some tumors remain asymptomatic, others cause severe disability. Early detection and treatment can prevent irreversible damage and improve the quality of life^[20,21]^.

### Conventional treatment options

Conventional treatment methods largely consist of surgery, radiotherapy, and, less frequently, chemotherapy. Each approach has its advantages and disadvantages.

### Surgery

Surgical resection is still the gold standard for meningioma, particularly resectable tumors. The Simpson grading scale (I–V) dictates the degree of resection and has a direct relationship with the recurrence rate. Total removal significantly reduces the recurrence rate, but skull base meningiomas are challengingly difficult to resect totally, and this raises the possibility of complications^[22]^. Despite the advantages of total surgical resection, there is controversy over the balance between aggressive tumor resection and patient morbidity. This is especially true in skull base meningiomas, which tend to be close to critical neurovascular structures^[23]^. Wide resections, particularly in complex anatomy areas, can cause morbidities like damage to cranial nerves, cerebrospinal fluid leaks, and injury to vessels. Due to the risk of morbidity with aggressive surgery, some neurosurgeons have adopted a conservative policy by performing subtotal resection with the option of adjuvant radiation therapy as required^[24,25]^. The main controversy in surgical treatment is whether maximal safe resection is always attempted or if subtotal resection and adjuvant therapy are acceptable alternatives. This choice is based on factors such as tumor site, histological grade, and patient comorbidities. Due to the risk of recurrence, especially with higher-grade meningiomas, close postoperative follow-up by MRI is important for long-term care^[25–28]^.

### Radiation therapy

Radiation therapy is vital in the management of meningiomas, especially atypical tumors, or if surgery cannot be done. The two highly useful methods are fractionated radiation therapy (FRT) and stereotactic radiosurgery (SRS), which are chosen depending on the size, location, and grade of the tumor^[29,30]^. FRT is majorly indicated for large or high-risk tumors, especially WHO Grade II and III meningiomas following subtotal resection^[31,32]^. SRS is well indicated for small or inaccessible tumors or recurrent tumors, therefore minimizing the recurrence rate^[33]^. However, there are still issues like standardized dosing for recurrences, long-term toxicity risks such as edema and cognitive impairment, and low efficacy in high-grade meningiomas^[34]^. Radiation may decelerate tumor growth but is inadequate for WHO Grade III meningiomas, which may require combined treatments. Tailored radiation plans and new drug combinations can potentially improve results. With all its disadvantages, radiation remains a central component of meningioma therapy, especially when surgery is not possible, with improvements aimed at further maximizing efficacy with less harm^[32]^.

### Chemotherapy

Chemotherapy has shown limited efficacy in meningioma treatment, as most tumors are slow-growing and resistant to conventional cytotoxic therapies^[35]^. Still, some chemotherapeutic agents have been used for recurrent or high-grade lesions. Hydroxyurea has been explored for its anti-proliferative properties and has shown some efficacy in controlling tumor growth, particularly in patients who are not candidates for surgery or radiation^[36]^. Temozolomide (TMZ) has shown variable benefits in treating meningiomas, particularly in high-grade and recurrent cases^[37]^. The biggest debate about using chemotherapy for meningiomas is the lack of strong clinical evidence supporting its effectiveness. Unlike gliomas, meningiomas don’t respond well to traditional chemotherapy. As a result, researchers are focusing on targeted molecular therapies. Chemotherapy is usually reserved for aggressive or hard-to-treat cases. There’s a growing interest in developing treatments based on genetic and molecular changes, such as SMO and NF2 mutations^[12,38]^. Due to these limitations, chemotherapy is usually a last-resort treatment, with surgery and radiation being the main options. Continued research into targeted molecular therapies could provide better ways to control aggressive or hard-to-treat meningiomas.

**Figure 1.**
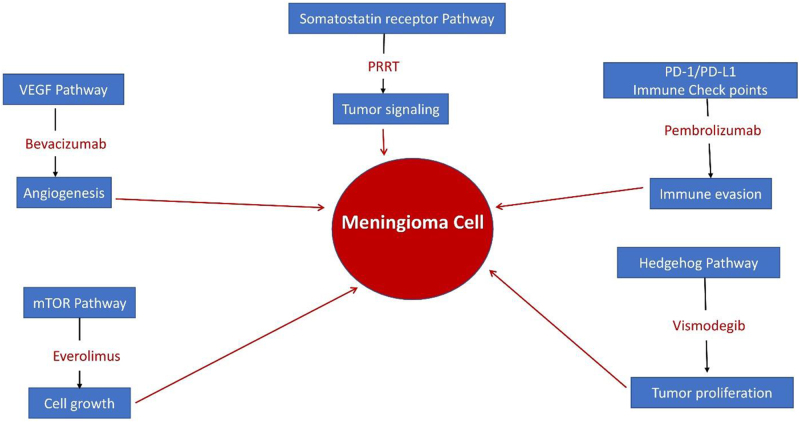
Key molecular pathways involved in meningioma growth, highlighting targeted therapies. In the figure, blue boxes represent the molecular pathways, while red text denotes the therapeutic agents that inhibit these pathways.

Figure 1 illustrates key molecular pathways involved in meningioma growth and progression, highlighting targeted therapies. Bevacizumab, a monoclonal antibody, inhibits VEGF-A, reducing tumor-associated vascularity. Everolimus, an mTOR inhibitor, slows tumor cell growth and is being investigated for patients with resistant meningiomas. Peptide receptor radionuclide therapy (PRRT) uses radiolabeled somatostatin analogs to disrupt tumor signaling, while Pembrolizumab blocks the interaction between PD-1 and PD-L1, enhancing the immune system’s ability to recognize and destroy tumor cells. Vismodegib, a hedgehog pathway inhibitor, targets and suppresses this pathway to limit tumor proliferation. Understanding these pathways offers new opportunities for developing personalized treatments, particularly for meningiomas that are challenging to manage through surgery or radiotherapy alone.

### Emerging treatment options

Advances in molecular profiling have paved the way for novel therapies that target specific genetic alterations in meningiomas, opening up potential new avenues of treatment. Targeted therapies present a promising strategy for managing recurrent meningiomas; however, the evidence remains inconclusive, with mixed outcomes highlighting their limited and variable efficacy across different cases.

### Targeted therapies

In this regard, Bevacizumab, a monoclonal antibody targeting vascular endothelial growth factor (VEGF), has been investigated as a therapeutic option for recurrent meningiomas, given the tumors’ reliance on angiogenesis.^[39]^ Clinical studies have yielded mixed outcomes regarding its efficacy. A multi-institutional phase II trial demonstrated that bevacizumab was well-tolerated and showed promise as a systemic treatment for patients with recurrent and refractory meningiomas.^[40]^ However, a retrospective clinical study reported that while bevacizumab improved progression-free survival (PFS) and overall survival (OS) at 12 and 36 months post-surgery in patients with high-grade meningiomas, these benefits did not extend to 60 months, indicating a potential limitation in long-term efficacy.^[41]^. Additionally, a systematic literature review highlighted that although bevacizumab could stabilize disease progression in treatment-refractory meningiomas, the overall survival benefits remained inconclusive.^[42]^. These mixed results underscore the need for further research to identify patient subgroups that may benefit most from anti-angiogenic therapies and optimize treatment protocols for recurrent meningiomas.

Building on the exploration of anti-angiogenic therapies, mTOR inhibitors have also gained attention as a potential therapeutic approach for managing recurrent meningiomas. Recent studies suggest that mTOR inhibitors such as everolimus may effectively reduce tumor growth in resistant cases.^[43]^. The combination of the mTOR inhibitor everolimus and the somatostatin agonist octreotide has shown promise in treating aggressive meningioma. The CEVOREM phase II study showed that this combination resulted in a 55% survival rate at 6 months in patients with relapsed meningiomas.^[44]^. These findings highlight the potential for combination targeted therapies and the importance of genetic profiling for treating meningioma. Inhibition of the mTOR pathway in meningiomas may result in AKT compensatory activation, reducing therapeutic efficacy. This interaction between mTOR and AKT pathways complicates treatment strategies and highlights the need for a comprehensive understanding of how these pathways interact with each other.^[45]^. In addition, the lack of validated biomarkers for patient selection hinders approaches to individual treatment. MRI and histological examinations are standard diagnostic tests but are insufficient to accurately predict prognosis. The integration of molecular biomarkers, such as specific gene mutations, could improve risk stratification and guide treatment choices.^[46]^

### Immunotherapy

Similarly, immunotherapy has garnered significant interest as an emerging therapeutic approach for recurrent meningiomas. The efficacy of pembrolizumab, a PD-1 inhibitor, in patients with relapsed or remaining high-grade meningiomas has been studied in recent studies. The phase II study showed that pembrolizumab may be a viable treatment option in these patients^[47]^. The effectiveness of immunotherapy, including pembrolizumab, is correlated with tumor mutational burden (TMB), which is the number of tumor genome mutations. Higher TMB is usually associated with increased neoantigenicity, which may enhance the recognition and response of the immune system to tumor cells. However, the use of TMB as a predictive biomarker for the effectiveness of immunotherapy remains questionable. Some studies have shown that TMB does not consistently predict the response of patients to immunocompetent inhibitors^[48]^. This difference suggests that factors other than TMB may affect the treatment outcome and highlights the need for further research to identify reliable biomarkers for patient selection in immunotherapy. In addition, a phase II study is investigating the combined effect of stereotactic radiosurgery and pembrolizumab in the treatment of relapsed meningioma. The aim of this study is to assess whether this combination may increase therapeutic efficacy^[49]^.

### Peptide receptor radionuclide therapy (PRRT)

Shifting focus from immune-based approaches, Peptide Receptor Radionuclide Therapy (PRRT) has emerged as a distinct strategy for managing recurrent meningiomas. Peptide receptor therapy (PRRT) using somatostatin-targeted radionuclides such as Lu-177 DOTATE has proven to be a promising treatment for advanced meningioma. A phase II study presented at the annual meeting of the American Society of Radiation Oncology (ASTRO) in September 2024 showed that almost 80% of patients with refractory meningioma had no progression after 6 months of Lu-177 DOTATE treatment. However, the existing evidence is mainly based on small-scale studies. For example, a study in heavily treated patients reported a 57% cure rate after PRRT, which highlights the need for further research to confirm these results^[50]^. Although PRRT with Lu-177 DOTATE has shown promise in the treatment of advanced meningiomas in small-scale studies, only large, well-structured, multicenter studies can help determine and develop its true therapeutic potential and standard treatment protocols. Moreover, addressing the gap in the development of biomarkers will also be essential to enhance personalized treatment approaches in this patient population.

### Novel drug delivery systems

Furthermore, intrathecal therapy and convection-enhanced delivery (CED) have emerged as promising delivery systems for the treatment of meningiomas. Intrathecal administration involves the delivery of the therapeutic substances directly into the cerebrospinal fluid, which may increase the distribution of the drug throughout the central nervous system. However, studies have shown a potential dose-related risk associated with intrathecal methotrexate and have highlighted the need for caution when using methotrexate^[51]^. CED is a technique that facilitates targeted local administration of drugs to the brain by crossing the blood-brain barrier. This involves stereotactic catheterization in an area of interest for direct infusion of a therapeutic product. This method was first studied in glioblastoma and demonstrated its potential to deliver high-dose therapies while reducing systemic toxicity^[52]^. However, its use in meningiomas is less well established, and further research is necessary to determine the effectiveness and safety profile in the treatment process.

In summary, the knowledge gap surrounding emerging targeted therapies remains a significant challenge in the treatment of recurrent meningiomas; however, with ongoing research, the future holds promising prospects for more effective management strategies. Table 1 shows ongoing clinical trials for meningiomas.

**Table 1:** Ongoing clinical trials for meningiomas

NCT No.	Study title	Phases	Study status	Intervention
NCT02648997	An Open-Label Phase II Study of Nivolumab or Nivolumab/Ipilimumab in Adult Participants With Progressive/ Recurrent Meningioma	Phase 2	Active not recruiting	Nivolumab, Ipilimumab, Nivolumab, External Beam R
NCT05425004	Cabozantinib for Patients With Recurrent or Progressive Meningioma	Phase 2	Recruiting	Cabozantinib
NCT05940493	Abemaciclib in Newly Diagnosed Meningioma Patients	Phase 2	Recruiting	Abemaciclib, Placebo
NCT06830356	Somatostatin Receptor PET Imaging to Guide Radiotherapy Dose Escalation in High Risk Meningiomas.	NA	Recruiting	Dose escalation of radiation therapy
NCT06126588	Combination of Everolimus and 177Lu-DOTATATE in the Treatment of Grades 2 and 3 Refractory Meningioma: a Phase IIb Clinical Trial	Phase 2	Recruiting	Everolimus
NCT04997317	Treatment of Recurrent or Progressive Meningiomas With the Radiolabelled Somatostatin Antagonist 177Lu-satoreotide	Early Phase 1	Recruiting	177Lu-DOTA-JR11, 177Lu-DOTA-JR11
NCT03631953	Combination of Alpelisib and Trametinib in Progressive Refractory Meningiomas	Phase 1	Recruiting	Trametinib, Alpelisibetinib
NCT02847559	Optune Delivered Electric Field Therapy and Bevacizumab in Treating Patients With Recurrent or Progressive Grade 2 or 3 Meningioma	Phase 2	Recruiting	Bevacizumab
NCT04082520	Lutathera for the Treatment of Inoperable, Progressive Meningioma After External Beam Radiation Therapy	Phase 2	Recruiting	Gallium Ga 68-DOTATATE, Lutetium Lu 177
NCT03604978	Nivolumab and Multi-fraction Stereotactic Radiosurgery With or Without Ipilimumab in Treating Patients With Recurrent Grade II-III Meningioma	Phase 1/Phase 2	Recruiting	Nivolumab
NCT06275919	Regorafenib for Recurrent Grade 2 and 3 Meningioma (MIRAGE Trial)	Phase 2	Recruiting	Regorafenib, Local Standard of Care
NCT03279692	Phase II Trial of Pembrolizumab in Recurrent or Residual High-Grade Meningioma	Phase 2	Active not recruiting	Pembrolizumab
NCT02523014	Vismodegib, FAK Inhibitor GSK2256098, Capivasertib, and Abemaciclib in Treating Patients with Progressive Meningiomas	Phase 2	Recruiting	Vismodegib, FAK Inhibitor GSK2256098, Capivasertib, Abemaciclib
NCT04501705	Apatinib in the Treatment of Recurrent Atypical/Malignant Meningioma in Adults	NA	Recruiting	Apatinib Mesylate

## Comparative discussion: old versus emerging therapies

### Efficacy

The two most common traditional treatments for meningiomas are surgical resection and radiation therapy. Surgery remains the most widely used traditional primary treatment for meningioma and has produced positive results in patients. However, complete removal is not always possible, particularly for tumors in areas of the brain that are difficult to remove, such as meningioma in the skull, or that have a high risk of recurrence, such as meningioma in tuberculosa. In these cases, adjuvant radiation therapy is used to increase local control. RT is appropriate for patients with WHO Grade II or III meningioma. The goal of radiotherapy is to reduce the ability of the tumor to spread and control its progression. With the advent of computer technology, radiotherapy has become more precise and more individualized^[53]^. Studies have shown that the combination of surgery and RT may increase the rate of progression-free survival, especially in higher-grade meningiomas. Emerging systemic treatments for meningioma, such as tyrosine kinase inhibitors, angiogenesis-targeting monoclonal antibodies, and immunotherapies, aim to achieve a systemic cure. However, there is currently a lack of long-term efficacy data, as most studies are early-stage studies with limited follow-up time. For example, agents such as sunitinib and bevacizumab have shown promising anti-tumor activity in small Phase II studies, but their long-term benefit has not been demonstrated. Similarly, immunosuppressive agents such as nivolumab and pembrolizumab are being studied, and early results indicate a clinically relevant response in a subset of patients^[8]^. However, comprehensive long-term data is not yet available. Ongoing clinical studies are still exploring these therapies, but until more extensive and long-term studies are completed, the durability and long-term efficacy of these therapies will remain uncertain.

### Toxicity

Although radiation therapy is effective, it still carries risks such as radiation necrosis, which destroys healthy tissue and may occur in the brain, the head and neck, and elsewhere in the body, and potential cognitive decline in patients, which causes progressive loss of ability to think, such as memory, learning, and focus. This study looked at 45 patients who had received fractional radiotherapy for meningioma. The study found that 56% had severe complications, including neuropsychological and neurological impairment severe enough to require hospitalization or a significant change in lifestyle. In addition, 75% of the subtotal resected and irradiated meningiomas recurred in the course of follow-up^[54]^. Further progress in RT techniques aims to minimize these adverse effects by targeting tumor tissues specifically. Emerging therapies such as immunotherapy may result in immune-related adverse events, including inflammation and autoimmune disorders. The safety profiles of these treatments are still being determined in ongoing clinical studies^[55]^. Targeted therapies may cause toxicities such as proteinuria, colitis, and thrombocytopenia^[56]^. Bleeding, cranial nerve disorder, and CSF fistula may be caused during surgical resection, whereas amenia and thrombocytopenia may be a complication of chemotherapy.^[57,58]^

### Cost and accessibility

The study found stereotactic radiosurgery (SRS) to be more cost-effective than surgical resection (Sr). The SRS had a lower average cost per patient (EUR 9,964) compared with the SR (EUR 11,647), which resulted in an increase in life years saved by 0.45 years.^[55]^ The median cost of chemotherapy administration worldwide was approximately USD 142 per hour, varying by region and by specific cost components^[56]^. In the USA, the cost of a cycle of bevacizumab (a monoclonal antibody) was approximately $10 458. The cost of nivolumab (an immunosuppressive) was $13 296 per cycle in the US^[57]^. Treatment with immune checkpoint inhibitors such as nivolumab and pembrolizumab may cost between USD 12 500 and USD 13 700 per month. These figures may vary according to healthcare systems, regional reimbursement, and the type of cancer^[58]^.

### Controversies

Traditional chemotherapy has only been shown to be effective against meningiomas. However, ongoing research into the molecular and genetic properties of these tumors may reveal new therapeutic targets and potentially redefine the role of chemotherapy. Drug treatment can only be used when surgical and radiotherapy strategies are no longer available, for example, in the case of recurrent or progressive meningioma. Chemotherapy and molecular target therapy, such as alkylating agents, tyrosine kinase inhibitors, endocrine agents, interferons, and inhibitors of targeted molecular pathways, are available for the treatment of non-benign meningiomas. Although many drugs have shown efficacy in preclinical studies and some clinical trials, no single effective drug has been identified in the various clinical trials^[59]^. One study looked at the combination of PRRT and fractionated EBRT in the treatment of advanced symptomatic meningioma. The goal of this approach is to increase radiation doses to the tumor while minimizing the exposure to surrounding healthy tissues. The combination demonstrated feasibility and tolerance, suggesting potential benefits over single-modality treatments^[60]^. An extensive review discusses the development and clinical application of PRRT, highlighting its role in targeting tumors overexpressing specific receptors, such as somatostatin receptors. It emphasizes the potential of PRRT to deliver high radiation doses directly to tumors, leading to significant tumor reduction^[61]^.

Table 2 compares conventional and emerging therapies for meningioma, focusing on efficacy, toxicity, and cost. Conventional therapies, like surgery and radiation, have long-term outcomes but limited efficacy against aggressive or atypical subtypes. Emerging therapies, like targeted therapies and immunotherapies, offer greater precision and individualized treatment. Conventional therapies have well-established side effects but are associated with higher systemic toxicity. The cost is generally more affordable, but prolonged use may increase long-term financial burdens.

**Table 2:** Comparison of conventional and emerging therapies for meningioma, focusing on efficacy, toxicity, and cost

Treatment type	Therapy	Efficacy	Toxicity	Cost
Conventional	Surgical resection	Gold standard for meningioma, particularly for resectable tumors	Bleeding, cranial nerve disorder, and CSF fistula	High
				Vuong *et al* (2013)
			Ahmeit *et al* (2021)	
		Simon *et al* (2024)		
	Radiotherapy	Effective for grade 11 and 111 meningiomas	Radiation necrosis and neuropsychological and neurological impairment such as cognitive decline	Moderate to high
				Vuong *et al* (2013)
		Wang *et al* (2024)		
			Mathiesen *et al* (2003)	
	Chemotherapy	Limited efficacy, used mostly for non-benign meningiomas	Amenia and leucopenia	Low
				Sohi *et al* (2021)
			Sager *et al* (2020)	
		Zhao *et al* (2020)		
Emerging	Immunotherapy	Effective for recurrent meningiomas	Inflammation and autoimmune disorders	Very high
				Xu *et al* (2024)
			Conroy *et al* (2022)	
		Brastianos *et al* (2022)^[47]^		
	Targeted	Effective for recurrent meningiomas	Proteinuria, colitis, and thrombocytopenia.	High
		Barresi *et al* (2011)	Shih *et al* (2016)	Verma *et al* (2018)

## Key gaps and unanswered questions

### Evidence gaps

Lack of randomized trials comparing emerging therapies to standard care: The rapid development of new cancer therapies requires rigorous testing against established therapies. However, there are no randomized controlled trials (RCTs) that directly compare these emerging treatments with standard care. This gap prevents the identification of real efficacy and safety profiles of new therapies. For example, although anti-angiogenic treatments have shown promise, the lack of reliable randomized clinical trials limits definitive conclusions on their relative effectiveness^[62]^. This review addresses the lack of high-quality evidence for the systemic treatment of meningioma. It underlines the limited efficacy of conventional therapies and highlights the promising antitumor activity observed in small Phase II studies with tyrosine kinase inhibitors and monoclonal antibodies. The article highlights the need for large prospective controlled studies to confirm these preliminary findings.^[8]^ The article discusses the problems in treating high-grade meningiomas and those that relapse after conventional treatment. It notes that traditional chemotherapy and other targeted therapies have not yet produced significant clinical benefits. The review advocates clinical trials that match patients to target substances according to tumor genetic subtypes, with the aim of a personalized approach to treatment.^[63]^

Limited data on combination therapies: Combination immunotherapy with radiotherapy was proposed to improve the response to the cancer. Preclinical studies indicate that radiotherapy may modulate the tumor microenvironment and may increase the efficacy of immunotherapeutic agents. However, clinical data to support such a combination is limited. The challenges include determining the optimal dose, sequencing, and management of potential synergistic toxicity. The comprehensive review highlights these problems and emphasizes the need for well-designed clinical studies to determine the safety and efficacy of these combinations.^[64]^ A Phase II study in patients with aggressive meningioma investigated the efficacy of the combination of the mTOR inhibitor everolimus and the somatostatin agonist octreotide in patients with meningioma. The study reported a 6-month progression-free survival rate of 55%, indicating a potential benefit of targeted combination therapy. However, the study did not examine the integration of immunotherapy with radiotherapy, which is an area that would require further investigation.^[65]^

Combinatorial drug screening identifies carfilzomib and enzalutamide for the treatment of aggressive meningiomas. Researchers performed an automated combinatorial drug screening using human meningioma cell lines and tumor organoids derived from patients. The study found that the combination of the protease inhibitor carfilzomib and the androgen receptor inhibitor enzalutamide is highly synergistic and induces apoptosis in tumor organoids. Although promising, immunotherapy agents were not included in this preclinical study, which highlights the limited data on immunotherapy-based combination therapy in meningioma.^[66]^

### Biological gaps

Poor understanding of resistance mechanisms in recurrent tumors: The recurrence of cancer often indicates the development of resistance to initial treatment. The underlying mechanisms of this resistance remain poorly understood, which hampers the development of effective second-line therapies. Research suggests that tumors can be modulated by different pathways, including alternative angiogenic signals, to evade treatment. A deeper understanding of these mechanisms is essential to develop treatments that can overcome or prevent resistance.^[67]^ A study published in the International Journal of Radiation Oncology, Biology, and Physics analyzed samples of patients with primary and recurrent meningioma and identified determinants of relapse and resistance to treatment. Research has shown that recurrent tumors are upregulated by the SUZ12 target genes and hypermethylate the genome extensively. These findings suggest that epigenetic alterations may be involved in the recurrence of meningioma and in resistance to treatment^[68]^.

Resistance to apoptosis in meningioma cells: The comprehensive review identified several molecular mechanisms that may contribute to resistance to apoptosis in meningioma cells: Wnt signaling pathway: This pathway negatively regulates apoptosis in meningioma cells. Inhibition of the Wnt-β catenin pathway induced apoptosis in these cells. Long noncoding RNA (ncRNA): NCRNA such as SNHG1 and LINC00702 affect apoptosis by altering the Wnt pathway. CD163 and CLincin6 (CLND6): Overexpression of CD163 and suppression of CLND6 are associated with decreased apoptosis and increased tumorigenicity. RAS pathway and let-7d microRNA: Changes in the RAS signaling pathway and decreased let-7d microRNA expression were associated with reduced apoptosis in meningioma cells. Together, these molecular factors contribute to the mechanisms of resistance seen in recurrent meningiomas.^[69]^

No validated biomarkers for treatment response (e.g., anti-angiogenics): The effectiveness of anti-angiogenic therapy varies between patients, and there are currently no validated biomarkers for predicting individual response to treatment. Identifying such biomarkers is essential for the development of personalized therapeutic approaches. Studies have examined possible candidates, including circulating endothelial cells and imaging techniques, but none of these have been universally accepted in the field of clinical practice. The review discusses the challenges of validation of biomarkers and the importance of integrating research on biomarkers in clinical trials^[70]^.

### Clinical gaps

Heterogeneous definitions of “progression” in trials: The heterogeneity of meningiomas leads to variability in prognosis. Progressive and recurrent meningioma (P&R) may occur with any grade of meningioma and is a common adverse outcome of surgical treatment and a major cause of post-operative re-hospitalization, secondary surgery, and mortality. Early prediction of (P&R). plays an important role in post-operative management, subsequent adjuvant therapy, and patient monitoring. Therefore, it is necessary to analyze the heterogeneity of meningiomas in detail and to predict post-operative PPR using non-invasive pre-operative imaging. In recent years, the development of advanced MR imaging technologies and machine learning has provided new insights into the non-invasive pre-operative prediction of meningioma P.R., which contributes to the accuracy of the prediction of meningioma P.R.^[71]^.

Underrepresentation of high-grade meningiomas in research: High-grade meningiomas (WHO grade II and III) present a significant clinical challenge due to aggressive behavior, higher recurrence rates, and worse prognosis than low-grade meningiomas. Despite these factors, research on high-grade meningiomas is still particularly limited, which leads to gaps in understanding their biology, response to treatment, and long-term outcome. This underrepresentation in scientific studies and clinical trials has a direct impact on the development of effective treatment strategies and standardized treatment protocols. One of the main reasons for this lack of research is the relative rarity of high-grade meningiomas as compared with tumors of grade I. Most of the meningioma cases diagnosed are benign (grade I), and consequently, research efforts and funding are often allocated disproportionately to the study of these minor tumors. Another critical issue is the heterogeneity of the high-grade meningiomas. These tumors have a variety of molecular and histopathological characteristics, which complicates the development of standardized treatment.

## Future directions

For physicians, meningiomas present a persistent clinical challenge. The subjective nature of WHO grading and the considerable variability in prognosis make it difficult to determine the most effective treatment strategy. The local therapeutic options are getting exhausted; there is an urgent need for novel treatment approaches to improve patient outcomes.Several ongoing clinical trials are investigating genetic alterations as potential therapeutic targets for meningiomas. One particularly intriguing target is the mTOR pathway, which is negatively regulated by NF2. Studies evaluating mTOR inhibition through Everolimus (NCT01880749, NCT01419639), Everolimus in combination with Octreotide (NCT02333565), and Vistusertib (AZD2014) (NCT03071874, NCT02831257) have demonstrated promising results in terms of tumor volume reduction and progression-free survival^[72,73]^.

For meningiomas with mutations in the Hedgehog (HH) pathway (SMO) or the NF2 pathway (FAK), targeted therapies such as vismodegib (an SMO inhibitor) and GSK2256098 (a FAK inhibitor) are being explored (NCT02523014). Additionally, a combinational approach utilizing the PI3K inhibitor Alpelisib and the MEK inhibitor Trametinib is under development for treatment-resistant meningiomas. While PI3K inhibition alone may not induce apoptosis – similar to mTOR inhibition – preclinical data from primary meningioma cell lines suggest that MEK inhibition can trigger caspase-mediated cell death. Therefore, this combined therapeutic strategy holds significant potential for improving clinical outcomes^[73]^.

Targeted radionucleotide therapy and somatostatin analogs, such as octreotide and pasireotide, have been explored with varying degrees of success due to the upregulation of somatostatin receptor 2A in many meningiomas. Additionally, a Phase 2 trial has demonstrated that the combination of octreotide and the mTOR inhibitor everolimus exhibits clinical activity and reduces tumor growth in WHO grade 1–3 meningiomas. Similarly, the progesterone antagonist Mifepristone has been investigated, along with other hormonal therapies, due to the high expression of progesterone receptors in meningioma cells. However, no clinically significant efficacy has been observed. Furthermore, tyrosine kinase inhibitors, particularly those targeting angiogenic pathways such as vascular endothelial growth factor (VEGF) signaling, have shown promising results. These findings suggest that VEGF-directed therapies may be beneficial in the management of higher-grade meningiomas. Supporting this hypothesis, a Phase 2 study of sunitinib in 36 patients with anaplastic and atypical meningiomas reported a progression-free survival (PFS) rate of 42% at 6 months (PFS-6). However, additional research is required before this approach can be established as a standard treatment for meningiomas. Moreover, DNA methylome analysis, which identifies biologically homogeneous subgroups, is increasingly being used as a supplementary diagnostic tool for CNS cancers. A comprehensive analysis of 497 meningioma samples has identified six distinct methylation clusters (benign 1–3, intermediate A/B, and malignant), which correlate with clinical factors such as sex, tumor location, and prognosis. In addition, certain meningiomas harbor mutations in genes such as KDM5C, KDM6A, SMARCB1, and SMARCE1, which encode proteins involved in transcriptional chromatin remodeling (SMARCB1, SMARCE1) or histone demethylation (KDM5C, KDM6A). These findings highlight the potential of epigenetic modifications as a novel therapeutic strategy for meningiomas^[8]^.

Assessing health-related quality of life (HRQoL) is crucial in evaluating the impact of treatments for meningioma patients. Studies have shown that patients with incidental and operated meningiomas found that both groups experienced impairments in HRQoL nearly a decade post-diagnosis or surgery. The study highlighted the importance of healthcare professionals being mindful of these impairments and directing patients to appropriate support^[74]^.

## Conclusion

The management of meningiomas is evolving with the introduction of novel therapeutic strategies. While surgery and radiotherapy remain the primary treatment options, their limitations, particularly in recurrent and high-grade cases, highlight the need for alternative approaches. The exploration of targeted therapies, immunotherapy, and innovative drug delivery techniques is reshaping treatment paradigms. However, despite encouraging preclinical and early clinical findings, the long-term efficacy and safety of these emerging therapies require further investigation. Immunotherapies, such as checkpoint inhibitors and peptide receptor radionuclide therapy (PRRT), have demonstrated potential, but their integration into routine clinical practice is still under evaluation.

A significant challenge in advancing treatment lies in the limited availability of randomized controlled trials comparing new therapies with conventional approaches. Many investigational treatments lack extensive clinical validation, making it difficult to assess their true therapeutic benefit. Additionally, combination strategies involving immunotherapy, chemotherapy, or radiotherapy require further optimization to enhance effectiveness while minimizing adverse effects. The absence of reliable biomarkers to predict treatment response further complicates the personalization of therapy.

A deeper understanding of the molecular and genetic alterations driving meningioma progression is essential for the development of more effective targeted therapies. Research into epigenetic modifications and key signaling pathways may lead to novel treatment options and better patient stratification in clinical trials. Future efforts should prioritize large-scale, multicenter studies to establish the safety and efficacy of emerging therapies while integrating precision medicine approaches.

Despite existing challenges, advancements in molecular profiling, targeted therapy, and immunotherapy offer promising avenues for improving patient outcomes. A multidisciplinary approach, combined with continued research and clinical innovation, has the potential to enhance the effectiveness of meningioma treatment and improve the quality of life for affected individuals.

## Data Availability

All data used in this narrative review are publicly available and sourced from previously published studies. No new data were generated for this work. All included articles have been appropriately cited within the manuscript and are available through the references section.

## Research registration unique identifying number (UIN)

Not required because it is a narrative review.
